# Avoiding perceived past resource use of potential competitors affects niche dynamics in a bird community

**DOI:** 10.1186/s12862-014-0175-2

**Published:** 2014-08-15

**Authors:** Jukka T Forsman, Sami M Kivelä, Tuomo Jaakkonen, Janne-Tuomas Seppänen, Lars Gustafsson, Blandine Doligez

**Affiliations:** 1 Department of Biology, University of Oulu, Oulu, FI-90014, Finland; 2 Department of Biological and Environmental Sciences, University of Jyväskylä, Jyväskylä, Finland; 3 Department of Ecology and Genetics/Animal Ecology, EBC, Uppsala Universit, Norbyvägen 18D, Uppsala, SE-752 36, Sweden; 4 Department of Biometry and Evolutionary Biology, CNRS, University of Lyon, Villeurbenne Cedex, F-69622, France; 5 Current address: Department of Zoology, Stockholm University, Stockholm, SE-10691, Sweden; 6 Department of Biology, Section of Ecology, University of Turku, Turku, FI-20014, Finland

**Keywords:** Species interactions, Social information use, Resource partitioning, Intra- and interspecific competition, Niche division, Nest-site selection, Cavity nesting birds, Parus, Ficedula

## Abstract

**Background:**

Social information use is usually considered to lead to ecological convergence among involved con- or heterospecific individuals. However, recent results demonstrate that observers can also actively avoid behaving as those individuals being observed, leading to ecological divergence. This phenomenon has been little explored so far, yet it can have significant impact on resource use, realized niches and species co-existence. In particular, the time-scale and the ecological context over which such shifts can occur are unknown. We examined with a long-term (four years) field experiment whether experimentally manipulated, species-specific, nest-site feature preferences (symbols on nest boxes) are transmitted across breeding seasons and affect future nest-site preferences in a guild of three cavity-nesting birds.

**Results:**

Of the examined species, resident great tits (*Parus major*) preferred the symbol that had been associated with unoccupied nest boxes in the previous year, i.e., their preference shifted towards niche space previously unused by putative competitors and conspecifics.

**Conclusions:**

Our results show that animals can remember the earlier resource use of conspecifics and other guild members and adjust own decisions accordingly one year after. Our experiment cannot reveal the ultimate mechanism(s) behind the observed behaviour but avoiding costs of intra- or interspecific competition or ectoparasite load in old nests are plausible reasons. Our findings imply that interspecific social information use can affect resource sharing and realized niches in ecological time-scale through active avoidance of observed decisions and behavior of potentially competing species.

## Background

Resource acquisition and thus division of niche space among coexisting species is strongly impacted by individual behavior, both on the short- and long-term. Behavioral plasticity may promote the evolution of permanent phenotypic changes in morphology, physiology, or life-history traits [1],[2] that can further redirect resource use. One major mechanism changing behavior, and potentially the direction of phenotypic shifts, is social information use, in which the decisions and performance of others are used to adjust one’s own decisions [3]. Social information use is a widespread phenomenon in the animal kingdom ranging from arthropods to primates and is used in many important decisions from foraging site selection to mate choice [4]-[6]. Social information use may also occur between species, as indicated by recent evidence [7]-[9]. However, very little is known regarding whether and how observed behavior or resource use of other species can cause shifts in resource use and niche, and how lasting such shifts can be.

The theory of species coexistence [10],[11] postulates that overlap in resource use with other species results in competition and, consequently, natural selection leads to divergence of traits affecting resource acquisition [12]-[14]. In contrast, models of interspecific social information use [7],[8],[15] predict a more diverse set of possible net effects of species interactions; the presence of species with shared resource needs can also result in facilitative effects. This is because the presence or performance of putative competitors can be used as a source of information to adaptively adjust individual decisions, which is expected to result in a trade-off between costs of competition and benefits of information use with increasing ecological similarity, spatial proximity or temporal synchronization [7]. In line with the predictions of social information use derived from intraspecific contexts, interspecific information use can result in copying and convergence of behavior [16],[17], but also active avoidance of the behavior of individuals that seem to have poor performance [6],[16],[18],[19] or to avoid confrontation with stronger competitors. Interspecific information use thus has potential to either increase or decrease resource use overlap among coexisting species but the time-scale and the ecological context over which such shifts can occur is unclear.

The guild of cavity nesting birds consisting of resident tits (*Parus* and *Cyanistes* spp.) and migratory flycatchers (*Ficedula* spp.) in Europe has been a major model system for competitive species interactions. These species compete with each other [20],[21], and flycatchers have been suggested to both suffer from competition with tits [20]-[23] and benefit from their presence during breeding time [24],[25]. Recent studies have demonstrated that flycatchers are not only attracted to breed in the vicinity of tit nests [25],[26] but that they also selectively copy and reject novel, experimentally introduced nest-site feature preferences of tits [6],[16],[18],[19], depending on the perceivable fitness (clutch size) of the tits, potentially reflecting their individual quality and end result of the earlier decisions. These studies provide strong evidence about existence and effects of interspecific information use in animal communities, because nest site selection is an important [27],[28] and partially genetically determined [29] niche dimension in birds. However, two conditions must be met for shifts in resource use caused by the observed behavior of other species to have a long lasting effect on resource partition among species: (1) such shifts must be transmitted across time and (2) they should also affect resource use in the absence of the tutoring species.

Here, we used a long-term field experiment to investigate whether experimentally induced artificial species-specific nest-site feature preferences of three coexisting bird species portrayed by geometric symbols are transmitted across time and affect nest-site preferences in the subsequent breeding season. The experiment was performed in a community of three cavity nesting bird species, the resident great tit (*Parus major*) and blue tit (*Cyanistes caeruleus*), and the migratory collared flycatcher (*Ficedula albicollis*). All three species have overlap in resource use and enemies in terms of food, nesting sites, predators and parasites, and they are known to compete with each other with negative fitness consequences (see above). In this study, we created apparent, species- and patch-specific nest-site feature preferences by attaching geometric symbols on nest boxes for the breeding period depending whether the box was occupied by a tit (great or blue tit), a flycatcher, or was unoccupied. The function of this design was to create an appearance of novel, community-wide, species-specific nest-site feature preference at each forest patch, exhibited by all breeding birds and available nesting sites in a patch, and to examine whether the experimentally induced preferences affect decision-making in the subsequent year. The response was measured by monitoring the symbol choices of all individuals in the beginning of the next breeding season.

Within a single breeding season, great tits preferentially choose to copy the nest-site feature choices of conspecifics [30], while flycatchers prefer the apparent choices of tits [6],[16],[18],[19]. If these preferences extend across breeding seasons, choices of tits and flycatchers should converge on the symbols associated with tits. On the other hand, if antagonistic interactions are prevailing and shared preference entails net costs due to increased exploitation or interference competition [20],[26] avoidance of boxes with symbols previously associated to conspecifics and/or heterospecifics is expected. It is also likely that preferences differ between resident tits and migratory flycatchers because their interactions seem to be asymmetric, great tits suffer when breeding close to flycatchers [26] while flycatchers benefit from close association with tits [24],[25]. Finally, we expected that philopatric individuals, i.e. individuals that have bred in the experimental patches in the previous year, would show stronger responses than immigrant individuals, due to longer and stronger exposure, and thus higher information access, to the local species-specific symbol association.

## Results

### Species-level variation in symbol choice

During the four study years we altogether obtained 184 symbol choices by great tits, 219 by collared flycatchers, and 103 by blue tits. The symbol choices of the flycatchers (χ^2^ = 1.67, df = 2, P = 0.434) and the blue tits (χ^2^ = 0.08, df = 2, P = 0.962) did not differ from random. In great tits, however, 46.6% of the breeding pairs chose the symbol that was associated with an empty nest box in the previous year, which clearly differed from random expectation (χ^2^ = 15.38, df = 2, P < 0.0001). This trend also remained quite stable across study years. In 2007, 44.7% of the great tit individuals (17 choices out of 38) preferred the symbol associated with an empty nest box in the previous year, while the corresponding numbers in 2008 were 43.6% (24 out of 55), and in 2009 40.4% (23 out of 57). In 2010, the proportion of tits preferring symbol associated with an unoccupied nest box was somewhat higher (61.7%, 21 out of 34).

**Figure 1:**
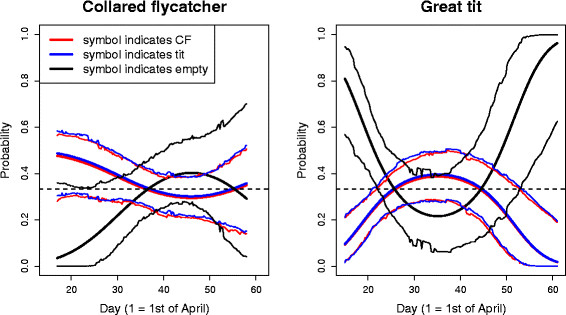
**The symbol preferences of the collared flycatcher and the great tit over elapsing time.** Fitted regression curves (thick lines) for each of the three symbols and their 95% highest posterior density credibility intervals (thin lines) in relation to the day of symbol choice for both collared flycatchers (left) and great tits (right). The horizontal dashed line indicates a probability of 1/3, which is expected if symbol choice is random.

**Figure 2:**
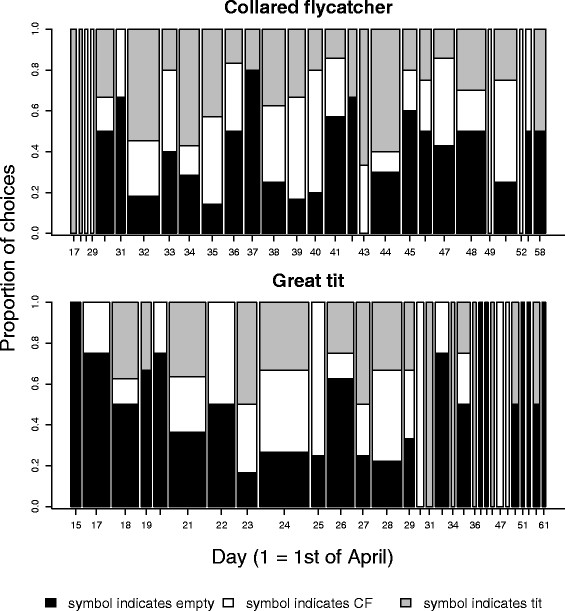
**The daily distribution of the collared flycatcher and great tit symbol choices across the settlement period.** The proportions are shown for each day when at least one pair chose their nest-site. Increasing width of a bar indicates more choices on that particular day, but the bar widths are not in the same scale in the two panels. The scale of the x-axis, day of symbol choice, refers to running day starting from the 1^st^ of April.

Identities of both pair members were known for 122 great tit and 139 collared flycatcher choices. In these data, there was an interaction between species and the quadratic effect of the day of nest site choice (Table 1 and Additional file 1: Table S1). This interaction arose because, in the great tit, the probability to choose the symbol associated with unoccupied boxes in the preceding season differed from random both at the beginning and at the end of the season, but dropped in the middle of the season, whereas in the collared flycatcher, symbol choice did not differ from random over the whole season (Figures 1 and 2). In great tits, the symbol associated with unoccupied boxes was preferred, and the symbols associated with collared flycatchers and tits in the previous year were avoided, both at the beginning and at the end of the season (Figures 1 and 2). The results of the similar analysis including all great tit and flycatcher observations irrespective of whether we had exact information about their identity (see Methods) yielded almost identical results (Additional file 1: Tables S2, S3 and Figure S1) compared to those obtained with the more restricted and accurate dataset, indicating that the results are robust.

**Table 1:** Summary of the model explaining symbol preferences of the great tit and the collared flycatcher

**Parameter**	**Posterior mean**	**95% credibility interval**	
		**Lower bound**	**Upper bound**
**Trait (symbol indicates CF)**	−7.76	−12.8	−2.60
**Trait (symbol indicates tit)**	−7.74	−12.8	−2.70
**Selection day**	0.476	0.123	0.808
**Selection.day**^**2**^	−0.00677	−0.0121	−0.00216
**Species (CF)**	14.7	3.25	26.8
**Selection day × Species (CF)**	−0.793	−1.44	−0.205
**Selection.day**^**2**^ **× Species (CF)**	0.0102	0.00196	0.0181

Parameter estimates (posterior means) and their 95% highest posterior density credibility intervals of fixed effects of the final generalized linear mixed-effects model fitted by MCMC simulation to the data on collared flycatcher and great tit symbol choices. The parameter ‘trait’ denotes the response variables [i.e. the probabilities of choosing a symbol associated with collared flycatcher (CF) or tit nests] in this multinomial logistic regression model. This model was fitted to the data restricted to the 261 observations where individual identities were known. See Additional file 1: Table S1 for parameter estimates of the starting model. Random effects included female and male identities (ring numbers) by allowing different variances among individuals in different years, and box identities.

### Immigration status and symbol choice

**Figure 3:**
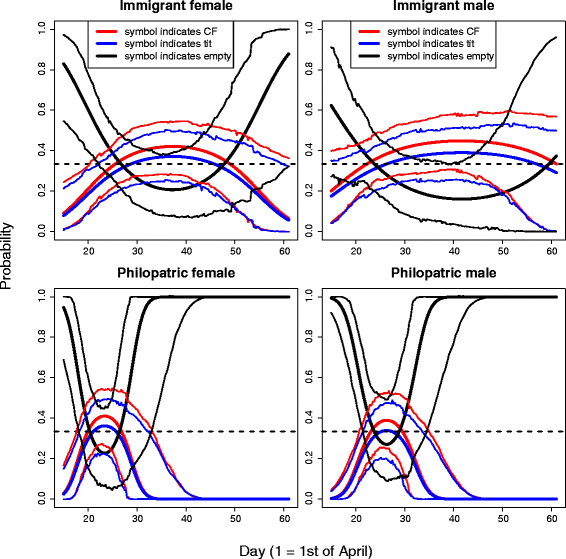
**The symbol preferences of male and female great tits with different immigration status.** Fitted regression curves (thick lines) for each of the three symbols and their 95% highest posterior density credibility intervals (thin lines) as a function of day of symbol choice for great tit females (left column) that were immigrant (top row) or philopatric (bottom row) to the habitat patch where they bred. The right column shows the corresponding results for immigrant (top row) and philopatric (bottom row) great tit males. The horizontal dashed line indicates a probability of 1/3, which is expected if symbol choice is random.

In great tits, the immigration status of both the female and the male affected symbol choice and the effect depended on the day of symbol choice (significant interactions between immigration status and day of nest site choice, Table 2 and Additional file 1: Figure S4). The symbol choice of immigrant great tit males was random throughout the season (Figure 3), while philopatric males strongly preferred the symbol associated to unoccupied boxes in the preceding year – and thus avoided the symbols associated with collared flycatcher and tit nests – both at the beginning and at the end of the season (Figure 3). Both immigrant and philopatric great tit females behaved very similarly to philopatric males as they also preferred the symbol associated to unoccupied boxes both at the beginning and at the end of the season (Figure 3), although the preference did not significantly deviate from random in immigrant females at the end of the season (due to wide 95% credibility intervals of symbol choice probabilities, Figure 3). Daily choices of nest-boxes and symbols for great tits with different immigration status are shown in Additional file 1: Figure S2. The immigration status affected symbol choice of neither female nor male flycatchers. The probabilities to choose any of the three symbols were in accordance with the random expectation in all cases (Additional file 1: Tables S5-S7).

**Table 2:** Summaries of the models explaining symbol preferences of the male and female great tits with different immigration status

**Sex**	**Parameter**	**Posterior mean**	**95% credibility interval**	
			**Lower bound**	**Upper bound**
**Male**	Trait (symbol indicates flycatcher)	−4.24	−9.77	2.00
	Trait (symbol indicates tit)	−4.38	−9.89	2.06
	Selection day	0.253	−0.152	0.611
	Selection.day^2^	−0.00303	−0.00806	0.00321
	Male status (philopatric)	−27.4	−50.3	−7.99
	Selection day × Male status (philopatric)	2.18	0.533	3.97
	Selection.day^2^ × Male status (philopatric)	−0.0432	−0.0792	−0.0115
**Female**	Trait (symbol indicates flycatcher)	−7.49	−13.8	−2.44
	Trait (symbol indicates tit)	−7.61	−12.9	−1.63
	Selection day	0.440	0.0968	0.789
	Selection.day^2^	−0.00590	−0.0108	−0.00107
	Female status (philopatric)	−23.8	−51.9	0.555
	Selection day × Female status (philopatric)	2.29	0.204	4.88
	Selection.day^2^ × Female status (philopatric)	−0.0524	−0.109	−0.00599

Parameter estimates (posterior means) and their 95% highest posterior density credibility intervals of fixed effects of the generalized linear mixed-effects model fitted by MCMC simulation to the data on great tit symbol choices including the effects of female and male immigration status (immigrant/philopatric). The parameter ‘trait’ denotes the response variables (i.e. the probabilities of choosing a symbol associated with collared flycatcher or tit nests) in this multinomial logistic regression model. See Additional file 1: Table S4 for parameter estimates of the starting models. Random effects included female and male identities (ring numbers) by allowing different variances among individuals in different years.

## Discussion

We experimentally demonstrated that great tits were able to remember and project the apparent nest-site feature preferences of conspecifics and other guild members in the previous breeding season onto nest-site choice one year after. Great tits that settled early or late strongly avoided the nest-site features associated with both tits and flycatchers and preferred the feature associated with unoccupied nest boxes. Such a shift towards apparently unused niche space was strongest among philopatric individuals, i.e. those which had bred in the same study plot in the previous year. The preference for the symbol associated with unoccupied nest boxes in immigrant females, which were not expected to respond to treatments, probably reflects male’s impact on nest-site selection decision [30] if they were mated with a philopatric male. These results emphasize that individuals can shift their resource use depending on the observed resource use of con- and heterospecifics. Importantly, this occurred without strong resource limitation (because there were plenty of empty nest-boxes available), indicating that social information use can have independent impact on resource use and niche dynamics within communities. In addition, most (c. 56%) great tits made their nest box choice before the first collared flycatchers returned from migration and initiated nest building, which demonstrates that they can actively avoid the features apparently preferred by flycatchers in the previous breeding season without flycatchers being present when the choice is made. Such information use, where decisions can be influenced without immediate association, may lead to more lasting and widely spreading shifts in resource use compared to a situation when imminent signal is needed to produce a response. Because our experimental design of using cross-controlled abstract geometric symbols as a substitute of nest-site characteristics effectively controls for innate or learned preferences, our results provide strong inference for the effects of social information use on resource use in animals.

Our experimental design cannot unequivocally distinguish whether great tits preferred for the unused niche space or avoided the preference of conspecifics and flycatchers. The avoidance of features preferred by conspecifics may reflect the costs of intraspecific competition and/or the avoidance of ectoparasites living in nest material. Classical niche theory predicts that niche width is a result of the expanding and reducing forces linked to intra- and interspecific competition, respectively [31]. Intraspecific competition can indeed expand the resource use of individuals in a population [32],[33] and changes can take place rapidly through behavioral plasticity as a response to resource availability [34]. Our results highlight that long-term evolutionary processes are not necessarily needed for niche shifts to occur (cf. [34]): the perceived resource use of con- and heterospecifics, even without strong resource limitation, can also trigger niche shifts within individuals’ lifetime. However, if the strategy of preferring previously unused resources is driven by reducing intra- and interspecific competition, its prevalence and benefits may depend on the population density. High population density, and in particular high number of philopatric individuals, may increase competition for previously unused resources. At low or intermediate densities, or if population includes a low proportion of philopatric individuals, a strategy of preferring previously unused resources may result in reduced competition over the resource. Great tits may also have avoided nest features associated to tits because potential heterospecific competitors, the blue tits [21], exhibited the same apparent preference for a given symbol as great tits. However, the effect of blue tits is plausibly minimal because great tits dominate blue tits in the selection of nest-sites [20]. Another plausible force driving niche shift in nest site selection could be the presence of nest parasites. Shift towards unused nesting resource may be reinforced by ectoparasite loads frequently present in old tit nests, which can decrease nesting success [35]. In our study, nest boxes were cleaned after each breeding period, so visual signals of the presence of old nests or ectoparasites could not be utilized directly by the individuals – instead, avoidance mechanism was indirect, via responding to a nest-site feature associated to con- or heterospecifics. Parasitism could be a main force selecting for the use of social information to avoid settling in a potentially previously occupied site.

The quadratic effect of day on great tit nest site feature choices remains unknown, but plausible explanation is the varying intensity of competition over high-quality nest sites over settlement period. During the peak of the settlement period, from late April to early May, time constraints and competition for the best nest sites [36] and mates are at their highest, which is probably strengthened by the appearance, and fast accumulation, of flycatchers. Conceivably, decisions are then likely to be based on more immediate factors such as the occupancy status and owners of neighbouring boxes. At the end of the breeding period, competition decreases again and may allow individuals to either use social information gathered in the previous or current year. The choices of philopatric and immigrant birds were distributed rather evenly over the season suggesting that it cannot explain the observed pattern.

Great tits may also have avoided symbols that were apparently preferred by collared flycatchers in the preceding breeding season. Avoiding the reciprocal negative effects of direct interspecific competition between our two study species may explain this result [21],[23],[24],[26] but see [37]. Apparent competition [38], driven by shared nest predators, has also been shown to be a strong selective force causing divergence in nest-site use in birds [27],[28]. Additionally, "information parasitism" of tits by flycatchers could also explain the great tit response. In the pied flycatcher (*Ficedula hypoleuca*), a closely related species to the collared flycatcher, individuals have been shown to prefer to breed in the vicinity of tit nests and thereby gain fitness benefits [25], while great tits suffer from the proximity of pied flycatchers in terms of reduced nesting success [26]. In addition, flycatchers can copy apparent novel nest-site feature preferences of tits [6],[16], implying that they may actively penetrate into the niche space of tits. Conceivably, counter-behaviors may have been selected in great tits to escape the negative effects of flycatchers by avoiding the nest site features that they apparently prefer. In line with this hypothesis, Loukola et al. [39] demonstrated that one function of the egg-covering behaviour with hair in Paridae during egg-laying can be preventing flycatchers to obtain the important clutch size information [6],[19].

Irrespective of the ultimate mechanism(s) leading to avoidance of apparent con- and heterospecific resource use, our results add support for earlier findings that, in addition to the usually considered “copying rules” [40], learning strategies based on actively avoiding others’ choices also exist [6],[18],[19]. Avoiding using the same resources than others may be adaptive if it reduces the costs of overlapping resource use, such as competition and indirect negative effects caused by shared predators and parasites (apparent competition), or if it allows selecting higher quality resources depending on the quality of the demonstrators [6].

Collared flycatchers and blue tits showed no response to the artificial nest site feature preference of con- and heterospecifics in this study, yet, these species are just as likely as great tits to be able to respond to the association between symbols and the occupancy status of the box, and more generally to use intra- or interspecific information [6],[16],[18],[19],[41]-[43]. The sample size for blue tits was perhaps too small to detect an effect. Flycatchers probably rely more on the up-to-date information provided by tits that already have initiated breeding activities by the time flycatchers arrive in their nest-site feature selection decisions cf. [16],[25] than one year old cues. A recent study also showed that collared flycatchers do use information from the previous breeding season in their small-scale nest-site selection [44]. Philopatric individuals preferred to breed at a site where conspecifics were breeding in the previous breeding season. Also a high breeding success of conspecifics increased the likelihood of settlement close to such a site [44]. Thus, in across-year information use, it seems that flycatchers prefer to use small-scale intraspecific spatial location information about nest locations in their nest box selection rather than relying on more large-scale information about nest-site niche preferences, which our symbols were reflecting.

To conclude, our experiment showed that the perceived resource use of con- and heterospecifics can influence the resource use of animals later on, even without any apparent resource limitation and in the absence of heterospecifics. This result complements our understanding of the division of resources among coexisting species by showing that social information use in the form of avoiding the resource use of others, both within and between species, may affect the realized niches of coexisting species. This may have implications for the rate of phenotypic change of coexisting species because the observed effect on resource use was parallel with the theoretical predictions of intra- and interspecific competition. Hence, information use in interspecific context may complement the evolutionary effects of competition, and enhance the speed of the niche divergence among species using overlapping resources.

## Conclusions

We made the nest-site choices of three cavity nesting birds visible to all members of the local breeding community by attaching abstract symbols on their nest-boxes for the breeding season, and we examined whether nest-site feature preferences (symbols on nest boxes) are transmitted across breeding seasons and affect nest-site preferences in the next breeding season. We show that the great tit preferred the symbol that had been associated with unoccupied nest boxes in the previous year, i.e., their preference shifted towards niche space previously unused by putative competitors and conspecifics. This result highlights that the perceived resource use of con- and heterospecifics can influence the resource use of animals later on, even without any apparent resource limitation and in the absence of heterospecifics.

## Methods

### Experimental design

The experiment was conducted on three discrete study plots (inter-plot distance 4–5 km) on the island of Gotland, Sweden, in 2006–2010. The average nest box density in the study plots was 4–5 boxes/ha, and the number of nest boxes per plot varied between 48 and 68, with usually 1/3 of the boxes being occupied by tits (mostly by great tits), 1/3 by flycatchers, and the rest unoccupied.

**Figure 4:**
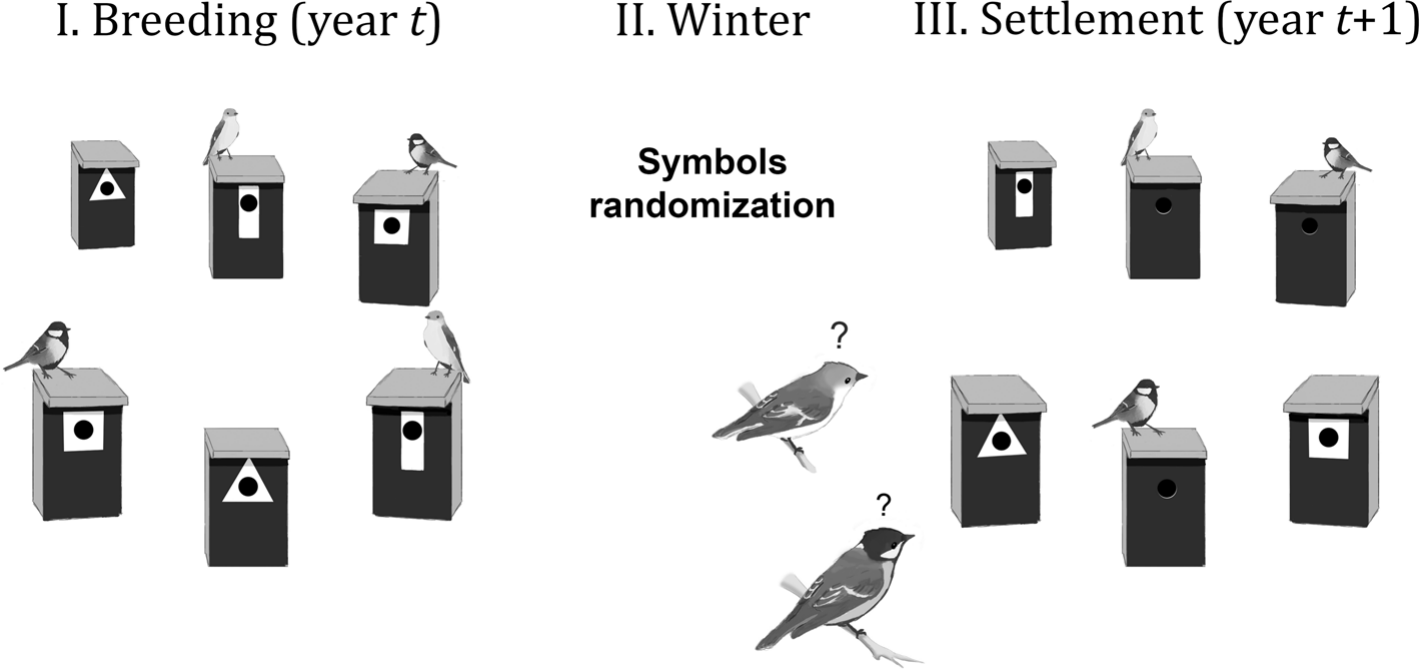
**Schematic presentation of the experimental design.** In step I, we created plot- and species-specific apparent symbol preferences during breeding period in year t. In step II, we randomised symbols on boxes after the breeding seasons in year t and year t + 1. In step III, the symbol preferences of resident tits and flycatchers in the experimental patches were monitored during the nest-site selection period. After each choice the symbol was removed from the box to exclude social information from the current spring for the subsequent birds. The proportions of the three symbol types during the spring were kept equal by switching symbols of empty nest boxes if needed.

The experiment consisted of three stages that were repeated each year during the experiment: (1) creating a plot- and species-specific apparent symbol preference during breeding period in year *t*, (2) randomizing symbols on boxes after the breeding season in year *t* and before the onset of the next breeding season (year *t* + 1), and (3) monitoring the symbol choices of all birds in early spring in year *t* + 1 (Figure 4). The experiment started in the beginning of June 2006 when all birds had initiated breeding. We attached white plastic geometric symbols (triangle, square, or rectangle) on boxes depending on whether the box was occupied by a pair of tits or flycatchers, or was empty (stage I). The front of each box was painted black before attaching the symbols to increase the contrast to the white symbol and its visibility. The species-specific apparent symbol preference was randomized and cross-controlled so that the symbol assigned to a particular box status (occupied by tits, occupied by flycatchers, or unoccupied) was different in each of the three study plots. The symbols assigned to each box status remained the same in each plot over the four years of the experiment. This procedure created an experimentally maintained appearance of local and long-term distinct species-specific nest-site feature preference, which both local breeders and prospecting individuals coming from elsewhere [45] can observe.

In the next winter, the old nests were cleaned from boxes, the symbols were removed and new symbols were randomly attached on the boxes (stage II). The frequencies of the symbols were set equal (1/3 of boxes with each symbol). Randomization allows controlling for the effect of the box or its surroundings (e.g., food resources, past occupancy) and earlier experience or acquired information [36],[41] on its probability to be occupied in the next spring.

In the following spring, the symbol preferences of all birds settling in the experimental patches were monitored during the nest-site selection period (stage III). We started monitoring nest boxes prior to the initiation of nest building of the first tits (early April) and continued until the last flycatchers had started breeding (early June). We checked all nest boxes every second day, and the choice of a box and a symbol together with the day of choice were determined by the detection of nest material in a box. Upon recording the choice, we removed the symbol so that later arriving individuals would not perceive the current-season symbol choices of previously settled individuals. Symbol frequencies were kept equal on the remaining vacant boxes (1/3 of each symbol) by changing symbols in case of over/under-representation of some symbols. This procedure ensured that any preference by the birds that could be detected could only result from the symbol-occupancy status associations observed in the previous year. After the nest-site selection period was over in the first days of June, symbols were again assigned to boxes occupied by tits, flycatchers or remaining unoccupied, according to the fixed local apparent species-specific preferences.

Great tit and flycatcher adults breeding in nest boxes were captured and ringed during incubation (flycatcher females) or nestling rearing (tits and flycatcher males) period in the study plots each year, and all nestlings were ringed. These data were used to categorise the captured birds as philopatric (if they bred in the same plot in the previous year) or immigrants (if they did not breed in the plot in the previous year), which plausibly can affect their knowledge about the local species-specific symbol associations and their subsequent symbol preferences.

### Statistical analyses

Statistical analyses were performed with R 2.15.1 [46]. We analysed the data in three steps. First, we tested whether the symbol choices deviated from random within each species (χ^2^ test, analysis I). For this analysis, we used all choices, i.e. all nests where birds initiated egg-laying, irrespective of the later fate of the breeding attempt.

In analysis II, we included only observations where both the male and the female of a breeding pair had been captured during the nestling period and thus their identities (ring numbers) could be determined. These data were smaller than in the first analysis due to breeding failures and missing information on either of the adults, and were restricted to collared flycatchers and great tits only because we do not have detailed ringing data on blue tits. In these species, we tested whether the probabilities that birds chose boxes with symbols associated with flycatcher or tit nests or unoccupied boxes differ from random (i.e., 1/3 for each symbol) by using generalized linear mixed-effects models (GLMM) within the Bayesian framework [function ‘MCMCglmm’ [47] that utilises Markov chain Monte Carlo (MCMC) methods]. We set the categorical symbol of the chosen nest box (symbol associated with collared flycatcher nests, tit nests or unoccupied boxes in the previous year) as the response variable, and used multinomial distribution for it (see Additional file 1). The starting model included as fixed effects species (collared flycatcher/great tit), the day of nest site (symbol) choice (April day, i.e., consecutive day numbering since 1^st^ of April; both a linear and quadratic effect to take into account potentially nonlinear effects) and the selected symbol (triangle/square/rectangle) to control for possible innate preferences for particular geometric shapes. In addition, the starting model included the two-way interactions between the variable ‘species’ and response variable (‘trait’ in MCMCglmm syntax), and the two-way interactions between the variable ‘species’ and each of the linear and quadratic effect of the day of nest site choice and the selected symbol. The day of initiation of nest building is an important covariate; it has been shown to affect the probability of symbol choices e.g., [16]. Because the data included multiple choices of some individuals that bred in the study area in different years, we set the identities (ring numbers) of both parents as random effects, and allowed variation among individuals to be different in different years in both females and males. Moreover, the data includes repeated observations on the same nest boxes, so we set also box identity as a random effect. We hierarchically reduced the fixed effects by removing non-significant terms. We determined significance on the grounds of highest posterior density credibility intervals (function ‘HPDinterval’ [48]) of the estimates (i.e., the 95% credibility intervals of ‘significant’ terms did not encompass 0).

We also tested the sensitivity of the results of the analysis II by repeating the analysis with the data including all 403 observations on collared flycatchers and great tits, irrespective of whether individuals were identified or not. In this analysis, we assumed that those individuals whose identities (ring numbers) were not known were represented in the data only once. Artificial identities were created for the unidentified individuals.

In the analysis III, we analyzed the effects of female and male immigration status on symbol choice probabilities. Because the inclusion of the immigration status variables in the models described above resulted in convergence problems and estimation uncertainty (model overparameterized), we analysed the immigration status effects separately for collared flycatcher and great tit females and males. The fixed effects initially included the main effects of ‘trait’ (refers to response in MCMCglmm syntax) and female or male immigration status, the interaction between ‘trait’ and ‘status’, the linear and quadratic effect of selection day and the interactions between ‘status’ and linear and quadratic effect of selection day. Female and male identities were set as random effects, variation among individuals being allowed to be different in different years in both females and males. Box identity could not be included as a random effect because it resulted in severe convergence problems in these models. We performed model selection as explained above.

In all GLMM analyses, we defined inverse Wishart prior distributions for the random effects (female, male and box identities) (see Additional file 1). We assessed the convergence of the MCMC chains by visual evaluation of the MCMC chain time series, supplemented by an autocorrelation analysis (Additional file 1).

In analyses II and III, we back-transformed the model linear predictors to the scale of observations (i.e. probabilities) by using the inverse of the (logistic) link function, and derived posterior distributions for the species- (or status-) specific fitted regression curves describing the probabilities to choose each of the three symbols in relation to the day of nest site choice. Then, we determined the 95% highest posterior density credibility intervals of the regression curves and based our inferences on them. We assessed the randomness of symbol choices by comparing the 95% credibility intervals of the regression curves to 1/3, which is the value expected if all three symbols have an equal probability of being chosen (symbol choice is random).

### Ethical note

Experimental procedure followed the national legislation of Sweden and birds were handled and ringed under a ringing license from Swedish Museum of Natural History for professor Lars Gustafsson (University of Uppsala, Sweden).

## Competing interests

We declare that we have no competing interests.

## Authors’ contributions

The experiment was designed by JTF, J-TS and BD, data was collected by JTF, TJ, BD, LG and analysed by SMK and JTF, and all authors contributed to writing of the manuscript. All authors have read and approved the final manuscript.

## Additional file

## Supplementary Material

Additional file 1: Supporting detailed information about statistical analyses and results of the analyses (Tables S1-S7 and Figure S1 and S2). Click here for file
