# Supplementary material for: Avoiding perceived past resource use of potential competitors affects niche dynamics in a bird community
Source: BMC Evol Biol. 2014 Aug 15;14:175. doi: 10.1186/s12862-014-0175-2 (PMC4236583; doi:10.1186/s12862-014-0175-2)
Supplement: Additional file 1: — Supporting detailed information about statistical analyses and results of the analyses (Tables S1-S7 and Figure S1 and S2). [file s12862-014-0175-2-S1.docx]

**Additional file 1**

Supporting detailed information about statistical analyses and results of the analyses.

Contents:

- Details of the statistical analyses.

- Tables S1 – S7 (supporting result tables).

- Figures S1 and S2 (supporting result figures).

**Details of the statistical analyses**

As the response variable includes three classes (analyses II and III), we modelled the probabilities to choose either the symbol that was associated with collared flycatcher nests (P[symbol = CF]) or the symbol that was associated with tit nests (P[symbol = T]) by using a multinomial distribution (the probability to choose the symbol that was associated with empty boxes is 1 – P[symbol = CF] – P[symbol = T]). Thus, the probability to choose a symbol associated with an empty box is redundant for the analysis and, hence, the model was essentially a bivariate model with P[symbol = CF] and P[symbol = T] being the response variables. In the great tit data, 26.1% and 16.7% of the males and females, respectively, included two or more observations of the same individual in different years. The corresponding proportions for male and female flycatchers were 24.0% and 15.0%, respectively. We report the results of statistical analyses with credibility intervals, a 95% credibility interval encompassing 0 corresponding to an absence of effect.

In the Bayesian analyses at stage II (analysis of overall responses in collared flycatchers and great tits), the prior distributions for the individual-specific random effects (female and male identities) were defined to be inverse Wishart distributions with **V** equal to a 4 x 4 diagonal matrix (corresponding to the four study years) where the diagonal elements were set to 1/9 and ν = 5. The box identity random effects were defined so that **V** = 1/9 and ν = 2. The prior residual covariance matrix was set as 1/[3(**I** + **J**)], **I** and **J** being 2 × 2 identity and unit matrices, respectively (Hadfield 2012). The residual covariance matrix was fixed to remain constant through the MCMC simulation.

In the Bayesian analyses at stage III (analysis of the effect of dispersal status on symbol choice), we defined inverse Wishart prior distributions otherwise similarly as at stage II, except that for both female and male identity random effects **V** was set to a 4 x 4 diagonal matrix, the diagonal elements being 1/6. No box identity random effects were included in the models at stage III, so the prior only included individual-specific (female and male identities) and residual parts.

In all stage II and III analyses, we ran the MCMC chains for 3.2 × 10^7^ iterations. The burn-in period consisted of 1.2 × 10^7^ iterations, and the remaining 2.0 × 10^7^ iterations were sampled with a thinning interval of 2.0 × 10^4^, resulting in posterior distributions of 1000 observations. We assessed the convergence of the MCMC chains by visually evaluating the parameter-specific MCMC chain time series, and by calculating autocorrelations for the stored parameter estimates with 20 lags (20 multiples of the thinning interval, the smallest lag being the thinning interval itself). All autocorrelations of fixed effects had absolute values smaller than 0.1 in the models including both collared flycatchers and great tits (we calculated 760 fixed effect autocorrelations for these four models in total). In these models, a maximum of three autocorrelations of the estimated variance components (9 variance components were estimated, i.e., 180 autocorrelations were calculated per model) per model had absolute values larger than 0.1, the strongest being 0.156 in absolute value. This was considered acceptable. In the analyses including immigration status, autocorrelations of parameter estimates were generally low; only four out of the 1040 (in total) fixed effect autocorrelations calculated for these eight models had an absolute value higher than 0.1, the strongest of these having an absolute value of 0.115, the corresponding numbers for the variance components being 5 out of 1280 (the strongest being 0.147 in absolute value). Because of both the low autocorrelations and the visual evaluation of the MCMC chains indicated convergence, the inferences based on the fitted models were considered reliable.

**Table S1** Starting model parameter estimates (posterior means) and their 95 % highest posterior density credibility intervals of fixed effects of the final generalized linear mixed-effects model fitted by MCMC simulation to the data on collared flycatcher and great tit symbol choices. The parameter ‘trait’ denotes the response variables [i.e. the probabilities of choosing a symbol associated with collared flycatcher (CF) or tit nests] in this multinomial logistic regression model. This model was fitted to the data restricted to the 261 observations where individual identities were known.

| **Parameter** | **Posterior mean** | **95 % credibility interval** | |
| --- | --- | --- | --- |
|  |  | lower bound | upper bound |
| **trait (symbol indicates CF)** | -8.77 | -15.2 | -3.75 |
| **trait (symbol indicates tit)** | -8.90 | -15.1 | -3.60 |
| **Selection day** | 0.511 | 0.173 | 0.890 |
| **Selection.day^2^** | -0.00724 | -0.0126 | -0.00241 |
| **Species (CF)** | 16.4 | 3.92 | 31.29 |
| **Selected symbol (square)** | 0.981 | -0.502 | 2.27 |
| **Selected symbol (triangle)** | 0.709 | -0.496 | 1.91 |
| **Trait (symbol indicates tit) × species (CF)** | 0.261 | -0.472 | 0.970 |
| **Selection day × Species (CF)** | -0.868 | -1.62 | -0.229 |
| **Selection.day^2^ × Species (CF)** | 0.0112 | 0.00298 | 0.0209 |
| **Selected symbol (square) × species (CF)** | -0.924 | -2.72 | 0.698 |
| **Selected symbol (triangle) × species (CF)** | -0.334 | -2.15 | 1.38 |

Random effects included female and male identities (ring numbers) by allowing different variances among individuals in different years, and box identities.

**Table S2** Parameter estimates (posterior means) and their 95 % highest posterior density credibility intervals of fixed effects of the final generalized linear mixed-effects model fitted by MCMC simulation to the data on collared flycatcher and great tit symbol choices. The parameter ‘trait’ denotes the response variables [i.e. the probabilities of choosing a symbol associated with collared flycatcher (CF) or tit nests] in this multinomial logistic regression model. This model was fitted to the data containing all of the 403 observations and containing missing individual identities. See table A3 for parameter estimates of the starting model.

| **Parameter** | **Posterior mean** | **95 % credibility interval** | |
| --- | --- | --- | --- |
|  |  | lower bound | upper bound |
| **trait (symbol indicates CF)** | -4.35 | -8.30 | -0.826 |
| **trait (symbol indicates tit)** | -4.33 | -8.10 | -0.649 |
| **Selection day** | 0.260 | 0.0364 | 0.516 |
| **Selection.day^2^** | -0.00405 | -0.00765 | -0.000586 |
| **Species (CF)** | 8.98 | 0.735 | 17.7 |
| **Selection day × Species (CF)** | -0.476 | -0.914 | -0.00979 |
| **Selection.day^2^ × Species (CF)** | 0.00643 | 0.000741 | 0.0122 |

Random effects included female and male identities (ring numbers) by allowing different variances among individuals in different years (note that artificial identities were created for individuals whose ring numbers were unknown), and box identities.

**Table S3** Starting model parameter estimates (posterior means) and their 95 % highest posterior density credibility intervals of fixed effects of the final generalized linear mixed-effects model fitted by MCMC simulation to the data on collared flycatcher and great tit symbol choices. The parameter ‘trait’ denotes the response variables [i.e. the probabilities of choosing a symbol associated with collared flycatcher (CF) or tit nests] in this multinomial logistic regression model. This model was fitted to the data containing all of the 403 observations and containing missing individual identities.

| **Parameter** | **Posterior mean** | **95 % credibility interval** | |
| --- | --- | --- | --- |
|  |  | lower bound | upper bound |
| **trait (symbol indicates CF)** | -4.51 | -8.21 | -0.968 |
| **trait (symbol indicates tit)** | -4.77 | -8.54 | -1.18 |
| **Selection day** | 0.266 | 0.0182 | 0.489 |
| **Selection.day^2^** | -0.00410 | -0.00793 | -0.000929 |
| **Species (CF)** | 8.96 | 1.15 | 17.7 |
| **Selected symbol (square)** | 0.199 | -0.709 | 1.06 |
| **Selected symbol (triangle)** | 0.400 | -0.439 | 1.28 |
| **Trait (symbol indicates tit) × species (CF)** | 0.465 | -0.465 | 1.05 |
| **Selection day × Species (CF)** | -0.470 | -0.893 | -0.0238 |
| **Selection.day^2^ × Species (CF)** | 0.00635 | 0.000251 | 0.0118 |
| **Selected symbol (square) × species (CF)** | -0.302 | -1.44 | 0.963 |
| **Selected symbol (triangle) × species (CF)** | -0.791 | -1.89 | 0.414 |

Random effects included female and male identities (ring numbers) by allowing different variances among individuals in different years (note that artificial identities were created for individuals whose ring numbers were unknown), and box identities.

**Table S4** Starting model parameter estimates (posterior means) and their 95 % highest posterior density credibility intervals of fixed effects of the generalized linear mixed-effects models fitted by MCMC simulation to the data on great tit symbol choices including the effects of female or male immigration status (immigrant/philopatric). The parameter ‘trait’ denotes the response variables (i.e. the probabilities of choosing a symbol associated with collared flycatcher or tit nests) in this multinomial logistic regression model.

| **Sex** | **Parameter** | **Posterior mean** | **95 % credibility interval** | |
| --- | --- | --- | --- | --- |
|  |  |  | lower bound | upper bound |
| **Male** | trait (symbol indicates flycatcher) | -4.37 | -10.3 | 0.996 |
|  | trait (symbol indicates tit) | -4.26 | -9.43 | 1.69 |
|  | Selection day | 0.254 | -0.106 | 0.619 |
|  | Selection.day^2^ | -0.00307 | -0.00867 | 0.00228 |
|  | Male status (philopatric) | -27.4 | -50.3 | -5.46 |
|  | trait (symbol indicates tit) × Male status (philopatric) | -0.744 | -1.88 | 0.427 |
|  | Selection day × Male status (philopatric) | 2.22 | 0.566 | 4.17 |
|  | Selection.day^2^ × Male status (philopatric) | -0.0441 | -0.0808 | -0.00913 |
| **Female** | trait (symbol indicates flycatcher) | -7.59 | -13.4 | -1.89 |
|  | trait (symbol indicates tit) | -7.65 | -13.4 | -1.83 |
|  | Selection day | 0.446 | 0.0762 | 0.788 |
|  | Selection.day^2^ | -0.00601 | -0.0105 | -0.000544 |
|  | Female status (philopatric) | -24.1 | -51.4 | -0.408 |
|  | trait (symbol indicates tit) × Female status | -0.285 | -1.46 | 0.919 |
|  | Selection day × Female status (philopatric) | 2.33 | 0.217 | 4.75 |
|  | Selection.day^2^ × Female status (philopatric) | -0.0536 | -0.107 | -0.00682 |

Random effects included female and male identities (ring numbers) by allowing different variances among individuals in different years.

**Table S5** Fitted probabilities (posterior means) and their 95 % highest posterior density credibility intervals (in parentheses) that collared flycatchers choose a particular symbol [associated to flycatcher nests, tit nests or empty boxes in the previous year] when the status of either the female or the male is taken into account. See Table A6 for parameter estimates of the statistical models.

| **Sex** | **Status** | **Symbol associated to** | | |
| --- | --- | --- | --- | --- |
|  |  | **Flycatcher nests** | **Tit nests** | **Empty boxes** |
| **Male** | Immigrant | 0.321  (0.222, 0.405) | 0.362  (0.269, 0.463) | 0.317  (0.205, 0.423) |
|  | Philopatric | 0.281  (0.187, 0.384) | 0.317  (0.214, 0.427) | 0.402  (0.248, 0.551) |
|  |  |  |  |  |
| **Female** | Immigrant | 0.307  (0.221, 0.395) | 0.348  (0.258, 0.444) | 0.345  (0.248, 0.452) |
|  | Philopatric | 0.299  (0.197, 0.409) | 0.340  (0.220, 0.459) | 0.361  (0.182, 0.529) |

**Table S6** Parameter estimates (posterior means) and their 95 % highest posterior density credibility intervals of fixed effects of the final generalized linear mixed-effects models fitted by MCMC simulation to the data on collared flycatcher symbol choices including the effects of female or male immigration status (immigrant/philopatric). The parameter ‘trait’ denotes the response variables [i.e. the probabilities of choosing a symbol associated with collared flycatcher (CF) or tit nests] in this multinomial logistic regression model. A 95% credibility interval encompassing 0 corresponds to an absence of effect. See table A7 for parameter estimates of the starting models.

| **Sex** | **Parameter** | **Posterior mean** | **95 % credibility interval** | |
| --- | --- | --- | --- | --- |
|  |  |  | lower bound | upper bound |
| **Male** | trait (symbol indicates CF) | 0.0164 | -0.505 | 0.612 |
|  | trait (symbol indicates tit) | 0.139 | -0.442 | 0.668 |
|  | Male status (philopatric) | -0.369 | -1.17 | 0.552 |
| **Female** | trait (symbol indicates CF) | -0.115 | -0.627 | 0.434 |
|  | trait (symbol indicates tit) | 0.0129 | -0.472 | 0.533 |
|  | Female status (philopatric) | -0.0558 | -1.02 | 0.913 |

Random effects included female and male identities (ring numbers) by allowing different variances among individuals in different years.

**Table S7** Starting model parameter estimates (posterior means) and their 95 % highest posterior density credibility intervals of fixed effects of the generalized linear mixed-effects models fitted by MCMC simulation to the data on collared flycatcher symbol choices including the effects of female or male immigration status (immigrant/philopatric). The parameter ‘trait’ denotes the response variables (i.e. the probabilities of choosing a symbol associated with collared flycatcher or tit nests) in this multinomial logistic regression model.

| **Sex** | **Parameter** | **Posterior mean** | **95 % credibility interval** | |
| --- | --- | --- | --- | --- |
|  |  |  | lower bound | upper bound |
| **Male** | trait (symbol indicates flycatcher) | 5.69 | -5.11 | 18.16 |
|  | trait (symbol indicates tit) | 6.08 | -4.64 | 18.7 |
|  | Selection day | -0.256 | -0.899 | 0.228 |
|  | Selection.day^2^ | 0.00268 | -0.00346 | 0.0101 |
|  | Male status (philopatric) | 9.29 | -19.2 | 39.1 |
|  | trait (symbol indicates tit) × Male status (philopatric) | -0.758 | -1.79 | 0.135 |
|  | Selection day × Male status (philopatric) | -0.481 | -2.03 | 0.920 |
|  | Selection.day^2^ × Male status (philopatric) | 0.00609 | -0.0116 | 0.0249 |
| **Female** | trait (symbol indicates flycatcher) | 6.03 | -6.46 | 15.8 |
|  | trait (symbol indicates tit) | 6.05 | -6.10 | 16.1 |
|  | Selection day | -0.267 | -0.747 | 0.322 |
|  | Selection.day^2^ | 0.00279 | -0.00439 | 0.00893 |
|  | Female status (philopatric) | 19.0 | -19.8 | 59.1 |
|  | trait (symbol indicates tit) × Female status (philopatric) | 0.391 | -0.687 | 1.53 |
|  | Selection day × Female status (philopatric) | -1.00 | -3.15 | 0.804 |
|  | Selection.day^2^ × Female status (philopatric) | 0.0128 | -0.00891 | 0.0396 |

Random effects included female and male identities (ring numbers) by allowing different variances among individuals in different years.

**Figure S1** Fitted regression curves (thick lines) for probabilities to choose each of the three symbols and their 95 % highest posterior density credibility intervals (thin lines) in relation to the day of symbol choice for both collared flycatchers (left) and great tits (right). The horizontal dashed line indicates a probability of 1/3, which is expected if symbol choice is random. These regression curves were derived from the model that was fitted to the data containing all of the 403 observations and thus containing missing individual identities.

**Figure S2** The daily distribution of great tit symbol choices with different immigration status across the settlement period. The proportions are shown for each day when at least one pair chose their nest-site. Increasing width of a bar indicates more choices on that particular day, but the bar widths are not in the same scale in the four panels. The scale of the x-axis, day of symbol choice, refers to running day from the 1^st^ of April.
